# Rapid onset lung squamous cell carcinoma with prominent peritoneal carcinomatosis and an eosinophilic leukemoid reaction, with coexistence of the BRAF V600E and oncogenic KRAS G12A mutations: A case report

**DOI:** 10.3892/ol.2014.2169

**Published:** 2014-05-22

**Authors:** BIN LI, JING CHEN LU, DAN HE, JUN WANG, HUI ZHOU, LIANGFANG SHEN, CHUNFANG ZHANG, CHAOJUN DUAN

**Affiliations:** 1 Institute of Medical Sciences, Key Laboratory of Cancer Proteomics of Chinese Ministry of Health, Xiangya Hospital, Central South University, Changsha, Hunan 410008, P.R. China; 2 Division of Oncology, Xiangya Hospital, Central South University, Changsha, Hunan 410008, P.R. China; 3 Department of Radiology, Xiangya Hospital, Central South University, Changsha, Hunan 410008, P.R. China

**Keywords:** lung squamous cell, BRAF V600E mutation, KRAS G12A, coexistence

## Abstract

Peritoneal carcinomatosis from lung cancer is rare, particularly from lung squamous cell carcinoma (LSCC). Concurrent somatic BRAF and KRAS mutations within the same tumor specimen have not been reported. The present study describes the case of a treatment-naïve LSCC patient with coexisting BRAF V600E and oncogenic KRAS G12A mutations in the primary lung lesion and the peritoneal metastases. The patient presented with prominent peritoneal carcinomatosis and an eosinophilic leukemoid reaction, but no respiratory symptoms. The patient succumbed 8 days after the onset of the condition due to rapid aggravation of the peritoneal carcinomatosis. To the best of our knowledge, this is the first study concerning the coexistence of BRAF and KRAS mutations in LSCC. Intensive activation of ERK was also observed in the primary lung lesion and the peritoneal metastases. Although the exact pathogenesis was unclear, the observations indicated that in the present study, the BRAF V600E and KRAS G12A mutations may have cooperate in inducing the malignant phenotype of LSCC. This case also highlighted the potential aggressive course and unusual pattern of spread of this specific dual-mutated tumor.

## Introduction

Lung squamous cell carcinoma (LSCC) is one of the most prevalent subtypes of lung cancer worldwide and its pathogenesis is closely linked with tobacco exposure. SCC classically arises in proximal segmental bronchi and extends into the parenchyma and bronchial lumen, producing obstruction with resultant atelectasis or pneumonia. Metastasis to the peritoneum from LSCC is rare and occurs with a poor prognosis (1). Mutations in KRAS and BRAF are present at the low frequencies of 6 and 2%, respectively, in LSCC, mostly occurring in current or former smokers (2–5). It has been reported that BRAF and KRAS mutations do not occur concomitantly within the same tumor, as simultaneous mutation in the same RAS/RAF/MEK/ERK signal pathway are redundant for the tumor cells (3,5). The present study describes the case of a treatment-naïve LSCC patient with coexisting BRAF V600E and oncogenic KRAS G12A mutations in the primary lung lesion and the peritoneum metastases, and with prominent manifestations of peritoneal carcinomatosis and an eosinophilic leukemoid reaction. This study was approved by the Ethics Committee of Xiangya Hospital (Changsha, China) and was performed according to the Declaration of Helsinki. The patient provided written informed consent.

## Case report

**Figure 1:**
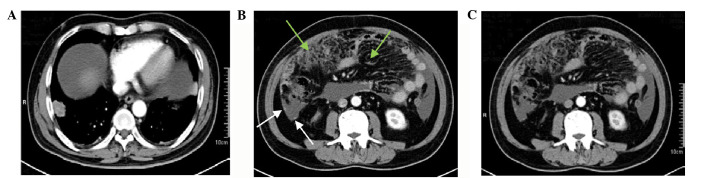
Computed tomography (CT) of the lungs and abdomen. (A) Primary lesion in the lower lobe of the right lung. (B and C) Caked colic omentum thickening (green arrow) and nodule-like thickening (white arrow) was more evident in the vein phase (B) than in the artery phase (C), as observed by CT with contrast enhancement.

**Figure 2:**
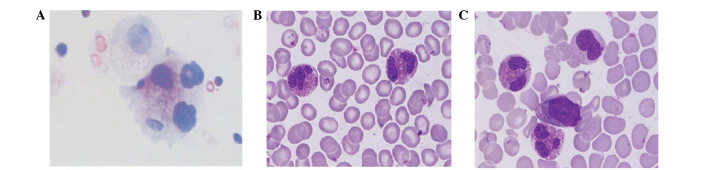
Cytology on ascites, peripheral blood and bone marrow. (A) Dyskaryotic cells in ascites sediment. (B) Representative peripheral blood smear and (C) bone marrow with eosinophilia.

**Figure 3:**
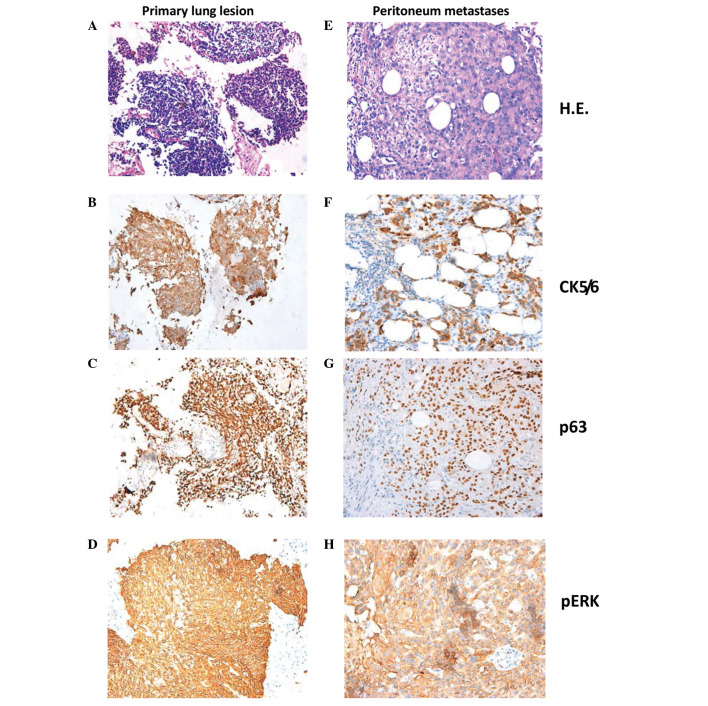
Hematoxylin and eosin staining and 3,3′-diaminobenzidine immunohistochemistry of the (A-D) primary lung lesion and (E-H) peritoneal metastasis. (A and E) Hematoxylin and eosin staining. (B and F) Cytokeratin 5/6 (positive in cytoplasm). (C and G) p63 (positive in nucleus). (D and H) pERK (positive in cytoplasm and/or nucleus) (magnification, ×200).

**Figure 4:**
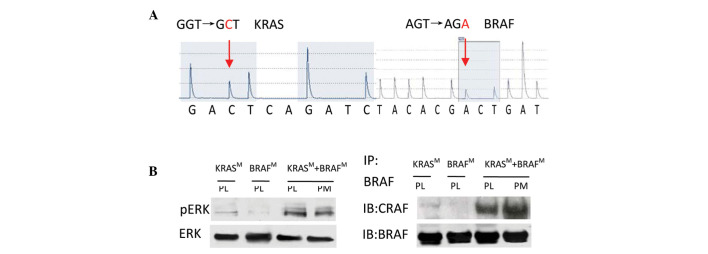
(A) Representive result from the gene sequencing of carcinoma tissue. (B) Cell extracts were prepared from carcinoma tissue and subjected to western blotting or immunoprecipitation using the indicated antibodies. KRAS^M^, a case with the KRAS G12A mutation; BRAF^M^, a case with the BRAF V600E mutation; PL, primary lung lesion; PM, peritoneal metastasis; IP, immunoprecipitation; IB, immunoblot. The data are representative of three independent experiments.

A 63-year-old male, with a 40-year smoking history (40 cigarettes per day) and a 35-year history of coal exposure, presented with intolerable abdominal distention, severe fatigue, adynamic fever and night sweats. These symptoms also dominated the clinical course of the disease. Computed tomography (CT) identified extensive, caked and nodule-like colic omentum thickening and massive ascites with a 2.9×2.5-cm primary lesion in the lower lobe of the right lung (Fig. 1). The sediment cytology of the ascites following centrifugation showed a number of dyskaryotic cells, indicating malignant ascites (Fig. 2). The blood and bone marrow cytology results showed leukocytosis of 52.2×10^9^/l, with 12.9×10^9^ eosinophils per liter. In total, 20% of the bone marrow cells were eosinophils (Fig. 2). The levels of serum interleukin (IL)-5, immunoglobulin E and granulocyte-macrophage colony-stimulating factor were normal. Evaluations for allergies, infection and autoimmune and clonal mechanisms were negative. CT-guided needle aspirations from the primary lung lesion and the thickened omentum were executed, and the histology from these aspirations revealed poorly-differentiated squamous cells, as confirmed by the cell morphology observed by hematoxylin and eosin staining, together with the positive staining for cytokeratin 5/6 and p63 and the negative staining for thyroid transcription factor-1 and napsin A observed by immunohistochemistry (Fig. 3). The biopsy specimens were sent for molecular analysis using tissue DNA extraction and pyrosequencing methods, which confirmed the tumor to be wild-type for EGFR exons 18, 19, 20 and 21, ROS1 and PI3K gene, and showed no abnormal ALK fusions. The presence of the BRAF V600E and KRAS G12A mutations was confirmed in the primary lung lesion and in the peritoneal metastases (Fig. 4A). Additionally, more excessive phosphorylation of ERK protein compared with total ERK was observed in the aspiration samples than in primary lung lesions from LSCC with a single BRAF V600E or KRAS G12A mutation, when analyzed by immunohistochemistry and western blot analysis (Figs. 3 and 4B). Notably, when using the co-immunoprecipitation method, endogenous BRAF and CRAF dimerization was found to be significantly enhanced in the tumor cells from the primary lung lesion and the peritoneum metastases in the present case (Fig. 4B). The prognosis was extremely poor, and the patient succumbed eight days after the onset of the condition due to rapid aggravation of the peritoneal carcinomatosis and disease resistance to treatment.

## Discussion

The present study reports a noteworthy case of peritoneal carcinomatosis from LSCC, with coexisting BRAF V600E and oncogenic KRAS G12A mutations in the primary lung lesion and peritoneal metastases. To the best of our knowledge, this is the first case of this type. Other clinicopathological features, including an eosinophilic leukemoid reaction, adynamic fever and rapid aggravation also make this case unusual.

LSCC typically presents with the local symptoms of coughing, chest pain and hemoptysis, or metastasis to the bone, liver, brain and adrenal glands. Peritoneal metastases, which usually originate from carcinoma of the gastrointestinal tract or ovary, are believed to originate from lung cancer rarely and from LSCC even more infrequently, always with a poor prognosis (1). Eosinophilia is rare and is usually due to IL-5 secretion in lung cancer, although the definitive cause in the present patient was unknown.

The patient of the present study primarily presented with combined somatic BRAF and KRAS mutations with an aggressive clinical history, unusual disease burden and poor prognosis. In animal experiments, oncogenic KRAS and activated BRAF mutations cooperate to accelerate the rapid onset of cancer (6). Oliveira *et al* (7) reported 5 cases of BRAF V600E and oncogenic KRAS that preferentially coexisted in advanced colorectal carcinoma; one of the 5 cases harbored the KRAS G12A mutation, indicating that activation of the two genes plays a synergistic role in tumor progression.

**Figure 5:**
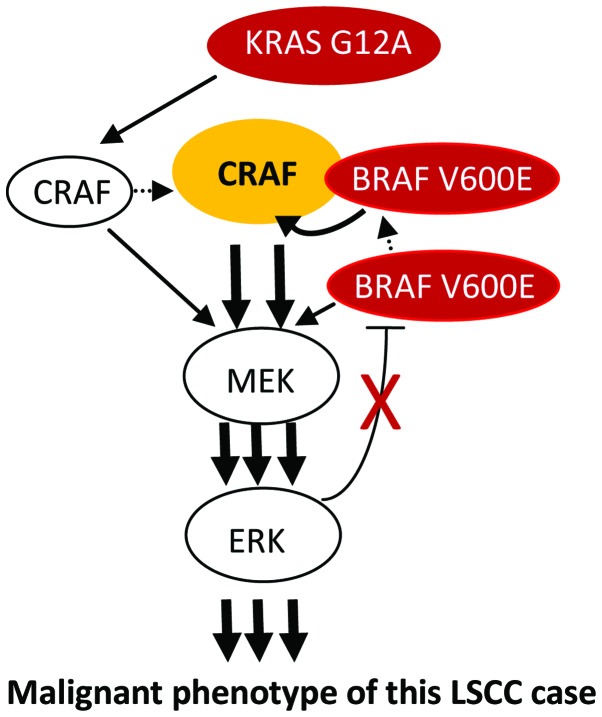
Possible model showing the cooperation of the activating mutations KRAS G12A and BRAF V600E in the present case of lung squamous cell carcinoma (LSCC). Simultaneous activation of the MEK/ERK signaling pathway by CRAF and BRAF occurs individually. BRAF V600E can transphosphorylate and hyperactivate CRAF in the presence of oncogenic KRAS, thus strongly augmenting MEK/ERK signaling activation. ERK-mediated negative feedback is highly resisted by BRAF-V600E.

The BRAF and KRAS molecules share a common RAS/RAF/MEK/ERK signaling pathway, and the ERK kinase is the downstream convergence point of the BRAF and KRAS signaling proteins (7). The present study therefore evaluated the phosphorylation level of ERK by immunohistochemistry and western blotting. The results showed that pERK expression, compared with total ERK, was strongly positive, and that it was more intense than the level in primary lung lesions from LSCC with a single BRAF V600E or KRAS G12A mutation. BRAF V600E is activated 500-fold more than the wild-type BRAF and directly phosphorylates the ERK signaling protein in cells, which plays a dominant role in promoting angiogenesis during tumor development. Oncogenic KRAS binds and activates CRAF more efficiently and mediates KRAS signaling to ERK kinase in lung cancer cells, thus fulfilling an important role as an anti-apoptotic protein independent of BRAF (6,8). Furthermore, BRAF V600E binds, transphosphorylates and hyperactivates CRAF in the presence of oncogenic KRAS to augment MEK/ERK signaling activation (9). The present study results showed that endogenous BRAF and CRAF dimerization was significantly enhanced in the tumor cells from the primary lung lesion and the peritoneal metastases in this case. These results raise the possibility that BRAF V600E cooperated with KRAS G12A to augment the ERK kinase activation, which may also have preference to eosinophil production. In addition, BRAF V600E is highly resistant to the two negative-feedback loops of ERK-mediated feedback phosphorylation and the induction of Sprouty proteins by ERK signaling via a delayed feedback loop (9), which may result in persistent and excessive activation of ERK kinase. One possible model showing the cooperation of the activating mutations, KRAS G12A and BRAF V600E, is shown in Fig. 5.

The occurrence of carcinoma is a multistep procedure and the result of an accumulation of gene mutations or abnormal expression. Cigarette and coal dust have been proven to be mutagenic factors of KRAS and BRAF in lung cancer (3). The present patient had an unusual history of heavy smoking with 40 cigarettes per day for 40 years and a 35-year history of coal exposure, which maybe the principal cause of the individual genotype found. A recent study has suggested that the dominant molecular oncogenes are associated with different biological behaviors manifesting as distinct patterns of metastatic spread in treatment-naïve lung cancer (10). Although the exact interaction between tobacco, coal and the carcinogenesis of bronchial epithelial cells has not yet been studied, the present case appears to indicate that intensive phosphorylation of ERK kinase caused the unusual spread pattern and eosinophilia of this specific type of LSCC, with dual mutations of KRAS G12A and BRAF V600E.

In conclusion, this is the first case study on the coexistence of the KRAS G12A and BRAF V600E mutations in LSCC. This case indicates that this specific double mutation of BRAF V600E and KRAS G12A results in a poor clinical course, potentially through the acceleration of tumor progression. The case also demonstrated an unusual spread pattern of this specific dual-mutated tumor, such that clinicians should be aware of its identification. Recently, certain studies have confirmed that single target inhibitors in a double BRAF-V600E and oncogenic RAS mutation accelerate tumor progression (11,12). Although the exact pathogenesis remains uncertain, the present study highlights the importance of obtaining a complete understanding of how networks function in this specific dual-mutated tumor. The mechanism of cooperation of the BRAF V600E and oncogenic KRAS mutations may represent a model for the mutation of differing molecules in the same signaling pathway.
